# A new look at an old well-being construct: evaluating the psychometric properties of 9, 5, and 1-item versions of emotional exhaustion metrics

**DOI:** 10.3389/fpsyg.2023.1267660

**Published:** 2023-11-23

**Authors:** Caitlin L. Penny, Kathryn C. Adair, Allan S. Frankel, Michael W. Leonard, Joshua Proulx, Paul J. Mosca, J. Bryan Sexton

**Affiliations:** ^1^Duke University School of Medicine, Duke University Health System, Durham, NC, United States; ^2^Duke Center for the Advancement of Well-being Science, Duke University Health System, Durham, NC, United States; ^3^Department of Psychiatry, Duke University School of Medicine, Duke University Health System, Durham, NC, United States; ^4^Vizient Safe and Reliable Healthcare, Evergreen, CO, United States; ^5^Duke Network Services, Duke University Health System, Durham, NC, United States; ^6^Department of Surgery, Duke University School of Medicine, Duke University Health System, Durham, NC, United States

**Keywords:** burnout, emotional exhaustion, Maslach burnout inventory, wellbeing, physician wellbeing assessment, burnout metrics, emotional exhaustion metrics

## Abstract

**Objective:**

To compare the relative strengths (psychometric and convergent validity) of four emotional exhaustion (EE) measures: 9- and 5-item scales and two 1-item metrics.

**Patients and methods:**

This was a national cross-sectional survey study of 1409 US physicians in 2013. Psychometric properties were compared using Cronbach’s alpha, Confirmatory Factor Analysis (CFA), Exploratory Factor Analysis (EFA), and Spearman’s Correlations. Convergent validity with subjective happiness (SHS), depression (CES-D10), work-life integration (WLI), and intention to leave current position (ITL) was assessed using Spearman’s Correlations and Fisher’s R-to-Z.

**Results:**

The 5-item EE scale correlated highly with the 9-item scale (Spearman’s rho = 0.828), demonstrated excellent internal reliability (alpha = 0.87), and relative to the 9-item, exhibited superior CFA model fit (RMSEA = 0.082, CFI = 0.986, TLI = 0.972). The 5-item EE scale correlated as highly as the 9-item scale with SHS, CES-D10, and WLI, and significantly stronger than the 9-item scale to ITL. Both 1-item EE metrics had significantly weaker correlation with SHS, CES-D10, WLI, and ITL (Fisher’s R-to-Z; *p* < 0.05) than the 5- and 9-item EE scales.

**Conclusion:**

The 5-item EE scale was repeatedly found equivalent or superior to the 9-item version across analyses, particularly with respect to the CFA results. As there is no cost to using the briefer 5-item EE scale, the burden on respondents is smaller, and widespread access to administering and interpreting an excellent wellbeing metric is enhanced at a critical time in global wellbeing research. The single item EE metrics exhibited lower convergent validity than the 5- and 9-item scales, but are acceptable for detecting a signal of EE when using a validated EE scale is not feasible. Replication of psychometrics and open-access benchmarking results for use of the 5-tem EE scale further enhance access and utility of this metric.

## Introduction

Healthcare worker (HCW) burnout is a global problem that negatively impacts wellbeing and quality of patient care, as highlighted by the COVID-19 pandemic (Shanafelt et al., 2010, 2019; Cimiotti et al., 2012; Kang et al., 2013; Welp et al., 2014; Wurm et al., 2016; Baier et al., 2018; Tawfik et al., 2018, 2019; Menon et al., 2020; Sexton et al., 2022b). Burnout is a complex construct with many contributing factors, thought to develop from an imbalance of high work demands and stressors (e.g., heavy workloads, difficult patient encounters, documentation requirements) with a lack of rewards and resources (e.g., helping others, recognition and income) (Demerouti et al., 2001; Lee and Mylod, 2019).

Healthcare worker burnout is associated with risks to patients through increased adverse events and patient mortality (Shanafelt et al., 2010; Cimiotti et al., 2012; Kang et al., 2013; Welp et al., 2014; Baier et al., 2018; Tawfik et al., 2018, 2019), and is associated with risks to the HCWs themselves through increased depression and suicidal ideation (Wurm et al., 2016; Menon et al., 2020). Burnout also increases financial costs for healthcare systems, physician turnover, and reduced productivity (Dewa et al., 2014; Shanafelt et al., 2016; Han et al., 2019; Adair et al., 2020b). With escalating demands on HCWs during the COVID-19 pandemic, concerns over the impact of burnout continue to increase (Bruyneel et al., 2021; Douglas et al., 2021; Haidari et al., 2021; Lasalvia et al., 2021; Macía-Rodríguez et al., 2021; Nguyen et al., 2021). As HCW wellbeing draws more attention and interventions are developed (Sexton and Adair, 2019; Adair et al., 2020b; Profit et al., 2021; Sexton et al., 2021b,2022a), the field suffers from a lack of consensus regarding rigorous assessment of “burnout,” which is evaluated using a variety of metrics and cutoff scores (Rotenstein et al., 2018).

After almost 50 years, the Maslach Burnout Inventory (MBI) is still considered the gold standard for assessing burnout among human services professionals, including HCWs (Maslach et al., 1997). It includes 22 questions and 3 subscales: emotional exhaustion (EE), depersonalization (DP), and personal accomplishment (PA). To date, EE is the most widely studied and reported component of the MBI and refers to being emotionally overextended and exhausted by the demands of work (Maslach et al., 2001). Compared to DP and PA, the 9-question EE subscale produces the largest and most consistent internal reliability estimates (Wheeler et al., 2011; Kleijweg et al., 2013; Loera et al., 2014), the highest test–retest reliability (Maslach et al., 1997), and has been demonstrated to be the only subscale adequately precise for individual-level measurement (Brady et al., 2020). EE also has the best ability to distinguish outpatients with an ICD-10 diagnosis of work-related neurasthenia or DSM-IV diagnosis of undifferentiated somatoform disorder, the closest clinical proxies to burnout (Schaufeli et al., 2001; Kleijweg et al., 2013).

Unfortunately, the length and cost of the MBI, which must be licensed and charges per use, limit its inclusion in health system surveys which often concurrently assess other important parameters (e.g., safety culture, engagement, and work-life integration) (Sexton et al., 2018). Assessing burnout rates and the impact of burnout interventions requires a valid, brief, and affordable metric that can be administered easily and quickly to busy HCWs while minimizing respondent burden. Longer surveys can cause fatigue among respondents and inaccurate results (Galesic and Bosnjak, 2009; Böckenholt and Lehmann, 2015; Liu and Wronski, 2018). As health systems increasingly focus on measuring and tracking burnout among HCWs, it can be a challenge to decide among the various metrics to administer, with several versions of the EE scale in particular, and a spectrum of cutoff scores used (Rotenstein et al., 2018). In order to facilitate efficient and accurate assessment of EE among populations of HCWs, abbreviated scales including 1-item (West et al., 2009; Li-Sauerwine et al., 2020) and 5-item (Sexton et al., 2018; Sexton and Adair, 2019; Profit et al., 2021) metrics have been developed. Notably, there are benefits to having scales with more than 1 item, including allowing for the assessment of internal reliability, a prerequisite of psychometrically valid assessments (Robinson, 2018).

A 5-item abbreviated version of the original 9-item EE scale has been carefully developed using items that are face valid and the scale has performed well psychometrically in prior research (Cronbach’s alphas ranging 0.84 to 0.92) (Adair et al., 2018, 2020a,2020c; Sexton et al., 2018; Schwartz et al., 2019; Sexton and Adair, 2019; Profit et al., 2021). It includes 4 items from the 9-item EE scale, in addition to a new item “events at work affect my life in an emotionally unhealthy way” (see Supplementary material for all items and response options). The response scale was also changed from a seven-point scale of frequency throughout the year ranging from “never” to “every day” (Maslach et al., 1997) to a five-point Likert scale ranging from “strongly disagree” to “strongly agree.” The transition from a seven-point to five-point scale was done to minimize respondent burden by maintaining consistency with other wellbeing and HCW-specific metrics that commonly use five-point scales (Sexton et al., 2006, 2018; Singer et al., 2007). Scales using responses on the agree-disagree spectrum can be applied to a wide array of constructs and are quickly and easily administered (Revilla et al., 2014). There is no significant difference in standard variation, skewness, or kurtosis between five-point and seven-point scales (Dawes, 2008), nor is there a difference in three key measures of response bias (Weijters et al., 2010). Despite the potential to increase reliability, validity, and discriminating power using seven-point response scales, the benefit of validated five-point scales is that they help to prevent respondents from being frustrated or demotivated, therefore improving response quality in settings of increased time constraint or longer surveys (Preston and Colman, 2000).

Given the importance of accurate and consistent EE assessment in contemporary healthcare, and the need for more clarity amidst various scales, items, response options and cutoffs, the current study aims to: (1) compare the psychometric properties of the 5-item, 9-item, and two 1-item EE metrics, and (2) assess the convergent construct validity of each EE scale with other important metrics used to assess wellbeing, including happiness, depression, work-life integration, and intention to leave current position. It is hypothesized that given the excellent psychometrics (Adair et al., 2018, 2020a,c; Sexton et al., 2018; Schwartz et al., 2019; Sexton and Adair, 2019; Profit et al., 2021) of the 5-item EE metric, it will have equivalent psychometric properties and higher convergent validity with the other metrics of interest relative to the 9-item, followed by the two 1-item metrics.

## Materials and methods

### Design and patient population

This study used cross-sectional survey data collected on a national sample of physicians, employed in a variety of hospital settings across the United States in 2013. Participants were members of a large healthcare system, contacted by email. They voluntarily responded to an anonymous electronic survey prior to initiating a continuing medical education (CME) activity to enhance wellbeing in clinical practice. In addition to emotional exhaustion, this wellbeing activity included assessments of happiness, depression, work-life integration, and intention to leave current position.

### Demographic information

The survey captured gender, years in in current position, years of work experience, shift type, and name of facility where the HCW was employed.

### Emotional exhaustion

Four measures of EE were captured in the survey: a 9-item, a 5-item, and two single item metrics. The 9-item EE scale from the MBI using a five-point Likert response scale (disagree strongly to agree strongly) was included (EE9item). The 5-item EE derivative of the 9-item version includes four items from the 9-item scale [(i.e., I feel…) (1) burned out, (2) fatigued, (3) frustrated, and (4) working too hard], and the additional item, “events at work affect my life in an emotionally unhealthy way” (EE5item). The EE5item has been tested in numerous large samples, has demonstrated good psychometric properties, and responsiveness to interventions (Adair et al., 2018, 2020a,c; Sexton et al., 2018; Schwartz et al., 2019; Sexton and Adair, 2019; Profit et al., 2021). Two single items of burnout were assessed. The first single item is “I feel burned out” using a five-point agree-disagree scale (EE1item5pt), and the second single item is “how often I feel burned out from my work” using a 7-point frequency response scale (EE1item7pt) (West et al., 2009). Table 2 lists the items included in each EE metric. For detailed information on scales, scoring, and benchmarking see the Supplementary material.

Responses to EE questions were averaged and rescaled from 0 to 100, with higher scores indicating more severe EE. Consistent with prior research, for five-point agree-disagree scales (EE9item, EE5item, EE1item5pt) a score of <50 indicates no EE (on average disagreeing slightly or strongly to all questions), 50–74 indicates mild EE (on average being neutral or agreeing slightly), 75–95 indicates moderate EE (on average agreeing slightly or strongly), and >95 indicates severe EE (agreeing strongly to all items). Scores ≥50 indicate concerning levels of EE, because the respondent is not disagreeing (i.e., neutral or higher) with the EE statements. To date, these cutoff scores have not been considered diagnostic, but rather to gauge the severity of EE present. The cutoffs provide an anchor for interpretation and a tool for communicating trends in the data. For the EE1item7pt metric, a cutoff of “once a week or more” is often used to indicate concerning levels (West et al., 2012; Brady et al., 2020; Li-Sauerwine et al., 2020). Responses to this item were also averaged and converted to a 0–100 scale to allow comparison to the five-point scales, with 66.67 on this scale indicating “once a week or more.”

### Depression

The Center for Epidemiological Studies Depression Scale 10-item (CES-D10) was used to measure depressive symptoms (Andresen et al., 1994). It uses a 4-point frequency scale with the prompt “during the past week, how often did this occur?” followed by phrases such as “I could not get going.” Responses were summed for a scale ranging from 0 to 30, with a score ≥10 considered a concerning frequency of symptoms (Björgvinsson et al., 2013).

### Subjective happiness

The Subjective Happiness Scale (SHS) is a validated, psychometrically sound, internationally used scale including 4 items on a seven-point scale, for example “In general I consider myself (1 = not a very happy person; 7 = a very happy person)” (Lyubomirsky and Lepper, 1999; Lin et al., 2010). Responses were averaged, with higher scores indicating higher subjective happiness. Prior studies have consistently demonstrated a mean SHS score of about 5 (Lyubomirsky and Lepper, 1999), so scores <5 were used to indicate concerning levels.

### Work-life integration

Items included in the measurement of work-life integration (WLI) are from the work-life climate scale (Sexton et al., 2017) using the prompt “during the past week, how often did this occur?” with a 4-point frequency scale ranging from “less than 1 day” to “5–7 days a week.” Examples include: “skipped a meal,” “arrived home late from work,” and “slept less than 5 hours.” Responses were averaged with higher scores indicating greater work-life imbalance. Consistent with prior research, mean scores >2, indicating average work-life imbalance >2 days a week, were used to indicate concerning WLB (Sexton et al., 2017; Schwartz et al., 2019; Tawfik et al., 2021).

### Intention to leave

To measure respondents’ intention to leave (ITL) their current position, 3 items using a five-point Likert scale ranging from “disagree strongly” to “agree strongly” were used (Sexton et al., 2019). Items included “I have plans to leave this job within the next year.” Higher intent to stay has been consistently shown to reduce job turnover (Brewer et al., 2012). Responses were averaged and converted to a scale ranging from 0 to 100, with higher scores indicating greater ITL. A threshold of ≥50 was used to indicate concerning ITL, as this is equivalent to responding “neutral” or higher, and not disagreeing with the questions.

### Statistical analysis

Descriptive analyses were performed for demographic variables. For all metrics, “not applicable” responses were treated as missing, Respondents who left 2 or more EE questions blank were excluded from all analyses. If 1 or more question was left blank on one of the other metrics (CES-D10, SHS, WLI, ITL), then a score for that metric was not included in the analyses. Cronbach’s alpha was used to measure internal reliability, assessing how closely related the set of questions are as a group. Alphas range from 0 to 1, with a reliability coefficient of at least 0.70 being acceptable for early stage research, 0.80 for implementing cutoff scores, and 0.90 for individual assessment or if clinically important decisions are being made (Nunnally, 1978; Nunnally et al., 1994). A unidimensional Confirmatory factor analysis (CFA) with maximum likelihood estimation was used to test how well the data fit the hypothesized underlying model construct of EE. The following fit indices were used: root mean square error approximation (RMSEA) with adequate fit <0.08, Tucker-Lewis fit index (TLI) with adequate fit >0.95, confirmatory fit index (CFI) with adequate fit >0.95, and standardized root mean square residual (SRMR) with <0.08 considered adequate fit (Browne and Cudeck, 1992; Hu and Bentler, 1999).

Analyses similar to prior research evaluating the EE1item7pt measure were also used (West et al., 2009). Spearman’s rank correlation coefficients were calculated to assess the convergent relationships among the different EE metrics and the SHS, CES-D10, WLI, and ITL metrics. This evaluates how well the relationship between two scales can be described using a monotonic function. Additionally, Spearman’s coefficients were calculated comparing the single item EE metrics to the EE scales while excluding shared items. To evaluate concordance of responses to the various EE scales, respondents were grouped based on their response to the EE1item5pt and EE1item7pt metrics, and mean EE9item and EE5item scores were calculated for those groups.

Convergent construct validity for each EE metric was assessed by comparing correlations to SHS, CES-D10, WLI, and ITL metrics. The correlations between each EE metric and the other metrics of interest were compared by converting correlation coefficients into a z-score using Fisher’s R-to-Z transformation then testing the difference between the two dependent correlations (Lee and Preacher, 2013). Additionally, the percent of respondents scoring above the concerning threshold for SHS (<5), CES-D10 (≥10), WLI (>2), and ITL (≥50) at various thresholds for severity of EE was calculated for all EE metrics.

Analyses were performed using JMP version 15.1 (SAS Institute Inc, 2019) and Mplus version 8.5 (Muthén and Muthén, 2010). The study was approved by the Duke University Health System Institutional Review Board (Pro00063703).

## Results

Of the 1836 physicians invited to participate, 1627 completed this survey (88.6% response rate). Of those, 120 (7.4%) did not consent to being in the research study and 98 (6.0%) left 2 or more EE questions blank and were excluded from analysis. Remaining results from 1409 participants were included in analyses. Participants reported working in over 200 different healthcare facilities and physician groups located in over 150 cities. Overall, 3.5% of participants did not report their clinical setting. The majority were male (64.4%). The most common responses for years in current position were 5–10 years (22.6%), 3–4 years (18.9%), and 1–2 years (15.6%). The most common responses for total years of professional experience were 21 + years (34.7%), 11–20 years (29.2%), and 5–10 years (21.8%). The majority worked day shifts (82.5%) with few working evening, night, or variable shifts (Table 1).

**TABLE 1:** Respondent demographics (*N* = 1409).

	*N* (% of total)
**Gender**	
Male	907 (64.4)
Female	501 (35.6)
Missing	1
**Years in position**	
<6 months	127 (9.0)
6–11 months	119 (8.5)
1–2 years	219 (15.6)
3–4 years	266 (18.9)
5–10 years	318 (22.6)
11–20 years	193 (13.7)
21 + years	166 (11.8)
Missing	1
**Years of professional experience**	
<6 months	21 (1.5)
6–11 months	15 (1.1)
1–2 years	56 (4.0)
3–4 years	107 (7.7)
5–10 years	305 (21.8)
11–20 years	409 (29.2)
21 + years	486 (34.7)
Missing	1
**Shift type**	
Days	1161 (82.5)
Evenings	11 (0.8)
Nights	10 (0.7)
Variable shifts	225 (16.0)
Missing	2

### Aim 1: compare the psychometric properties of the EE9item, EE5item, EE1item5pt, and EE1item7pt

After converting to a 0–100 scale, the mean scores and 95% CIs for the EE metrics in descending order are: EE5item 36.6 (35.2–38.0), EE1item5pt 34.3 (32.6–36.0), EE9item 31.6 (30.3–32.8), EE1item7pt 29.7 (28.4–31.0) (Table 2).

**TABLE 2:** Spearman’s correlation coefficients between EE, SHS, CES-D10, WLI, and ITL scales with correlations of unshared items in parentheses.

	EE9item: fatigued, burned out, frustrated, working too hard, emotionally drained, used up, at the end of my rope, too much stress, a strain for me	EE5item: fatigued, burned out, frustrated, working too hard, events at work	EE1item5pt: I feel burned out	EE1item7pt: How often I feel burned out from my work
EE5item	0.955 (0.828)			
EE1item5pt	0.861 (0.812)	0.878 (0.802)		
EE1item7pt	0.689 (0.671)	0.690 (0.663)	0.682	
SHS	−0.482	−0.493	−0.431	−0.389
CES-D10	0.575	0.568	0.504	0.508
WLI	0.464	0.476	0.373	0.380
ITL	0.569	0.588	0.516	0.433

For each pairwise correlation *p* < 0.0001. Values not in parentheses are Spearman’s correlation coefficients comparing the full scales to each other. Values in parentheses are Spearman’s correlation coefficients comparing only unshared items. For example, in the parentheses, the EE5item compared to the EE9item excludes the shared items (i.e., I feel fatigued, burned out, frustrated, working too hard), leaving only unshared items from the EE9item. EE9item, 9-item emotional exhaustion metric; EE5item, 5-item emotional exhaustion metric; EE1item5pt, single item emotional exhaustion metric using five-point agree-disagree scale; EE1item7pt, single item emotional exhaustion metric using 7-point frequency response scale; CES-D10, center for epidemiological studies depression scale 10-item; SHS, subjective happiness scale; WLI = work-life integration; ITL, intention to leave current position.

Both the EE9item and EE5item demonstrated excellent internal reliability with Cronbach’s alpha of 0.91 and 0.87, respectively. The CFA for the EE9item revealed poor fit with RMSEA = 0.219 (90% CI 0.211–0.228), CFI = 0.790, TLI = 0.720, and SRMR = 0.088. The CFA for the EE5item demonstrated improved fit with RMSEA = 0.082 (90% CI 0.063–0.103), CFI = 0.986, TLI = 0.972, and SRMR = 0.018.

Spearman’s correlation coefficient for the full EE5item and EE9item was rho = 0.956, (*p* < 0.0001). Spearman’s correlation coefficient for the EE5item and EE9item with the 4 shared questions excluded (I feel fatigued, burned out, frustrated, working too hard), was rho = 0.828 (*p* < 0.0001). Spearman’s correlation coefficient for the EE1item5pt and the EE9item with that question excluded (I feel burned out), was rho = 0.812 (*p* < 0.0001), and compared to the EE5item with that question excluded, was rho = 0.802 (*p* < 0.0001). When comparing the EE1item7pt the Spearman’s correlation coefficient for the EE9item excluding “I feel burned out” was rho = 0.671 (*p* < 0.0001), and compared to the EE5item excluding the burnout question rho = 0.663 (*p* < 0.0001) (Table 2).

To determine how the EE severity for a respondent would vary based on the EE metric used, all respondents who answered the same level of severity on the EE1item5pt and EE1item7pt (for example all respondents who select “agree strongly” to feeling burned out) were grouped. Then, the average EE scores on the EE5item and EE9item were calculated for each of those groups. This demonstrated that severe EE as assessed through single item EE metrics corresponded to lower EE using the EE9item relative to the EE5item. For example, using EE1item5pt, the average respondents who “agrees strongly” that they were burned out from work (score = 100/100), had an average response of only “agree slightly” for the EE9item (score = 74.4/100), and fell between slight and strong agreement for the EE5item (score = 86.1/100). For the EE1item7pt, there was a similar effect, with respondents who reported feeling burned out “every day” (score = 100/100) on the EE1item7pt only responding on average “once a week” to “a few times a week” (score = 76.1/100) on the EE9item (Table 3). In comparison, those who reported feeling burned out “every day” (score = 100/100) on the EE1item7pt, reported an average score of 88.6/100 on the EE5item.

**TABLE 3:** Translating single item EE scores to their corresponding 5 and 7 item scale scores.

EE1item5pt: “I feel burned out from my work” (numeric score out of 100)	*N* (%)	Mean EE9item score	Mean EE5item score
1. Disagree strongly (0)	503 (35.8)	9.8	11.3
2. Disagree slightly (25)	324 (23.0)	26.9	31.0
3. Neutral (50)	230 (16.4)	43.4	49.2
4. Agree slightly (75)	251 (17.9)	53.6	64.0
5. Agree strongly (100)	98 (7.0)	74.4	86.1
**EE1item7pt: “How often I feel burned out from my work” (numeric score out of 100)**	***N* (%)**	**Mean EE9item score**	**Mean EE5item score**
1. Never (0)	198 (14.1)	11.4	13.1
2. A few times a year or less (16.7)	614 (43.6)	21.8	25.4
3. Once a month or less (33.3)	221 (15.7)	35.4	41.0
4. A few times a month (50)	197 (14.0)	48.2	55.4
5. Once a week (66.7)	63 (4.5)	52.9	63.0
6. A few times a week (83.3)	84 (6.0)	67.6	78.8
7. Every day (100)	32 (2.3)	76.1	88.6

Possible EE scores range from 0 to 100. *N* missing for EE1item5pt = 3, for EE1item7pt = 0. EE9item, 9-item emotional exhaustion metric; EE5item, 5-item emotional exhaustion metric; EE1item5pt, single item emotional exhaustion metric using 5-point agree-disagree scale; EE1item7pt, single item emotional exhaustion metric using 7-point frequency response scale.

### Aim 2: assess the convergent construct validity of each EE metric for other important metrics assessing wellbeing including happiness, depression, work-life integration, and intention to leave current position

For completeness, Cronbach’s alphas were calculated for the other wellbeing metrics and were as follows: SHS = 0.83; CES-D10 = 0.80; WLI = 0.83; ITL = 0.87. Spearman’s correlation coefficients were calculated (Table 2) and used for the test of difference between two dependent correlations using Fisher’s transformation (Table 4). Results demonstrate that the EE5item is more strongly correlated with SHS, WLI, and ITL relative to the other EE metrics, but only significantly stronger than the EE9item for ITL. The EE9item and EE5item have significantly stronger correlations with all other metrics than the EE1item5pt and EE1item7pt. The EE1item5pt is significantly stronger correlated with SHS and ITL than EE1item7pt.

**TABLE 4:** Z-scores and *p*-values for two-tailed test of the difference between two dependent correlations for each EE metric and scores for SHS, CES-D10, WLI, and ITL using Fisher’s transformation.

	EE9item vs. EE5item		EE9item vs. EE1item5pt		EE5item vs. EE1item5pt		EE9item vs. EE1item7pt		EE5item vs. EE1item7pt		EE1item5pt vs. EE1item7pt	
	**Z-score**	***p***	**Z-score**	***p***	**Z-score**	***p***	**Z-score**	***p***	**Z-score**	***p***	**Z-score**	***p***
SHS	1.671	0.095	−4.08	<0.001**	−5.377	<0.001**	−4.998	<0.001**	−5.664	<0.001**	−2.209	0.027*
CES-D10	1.151	0.250	6.12	<0.001**	5.814	<0.001**	3.903	<0.001**	3.458	0.001*	−0.252	0.801
WLI	−1.695	0.090	7.237	<0.001**	8.774	<0.001**	4.514	<0.001**	5.186	<0.001**	−0.355	0.723
ITL	−2.876	0.004*	4.6	<0.001**	6.683	<0.001**	7.793	<0.001**	8.969	<0.001**	4.582	<0.001**

*P*-values indicated as * for < 0.01, and ** for < 0.001. Values larger than | 1.96| are considered significant using a two-tailed test, and the Z-score produced is negative if the first correlation coefficient entered into the formula is smaller than the second (Lee and Preacher, 2013). Values were entered in the order listed in the heading for each column (e.g., EE9item vs. EE5item results show no difference for SHS, CES-D10 and WLI, but that EE5item is *more strongly* associated with ITL). A positive Z-score indicates that the first item listed in the heading had a stronger correlation, except for SHS values which were negatively correlated with EE so the relationship is reversed. For the SHS values, a negative Z score indicates that the first EE metric in the heading is more strongly correlated. EE9item, 9-item emotional exhaustion metric; EE5item, 5-item emotional exhaustion metric; EE1item5pt, single item emotional exhaustion metric using 5-point agree-disagree scale; EE1item7pt, single item emotional exhaustion metric using 7-point frequency response scale; CES-D10, center for epidemiological studies depression scale 10-item; SHS, subjective happiness scale; WLI, work-life integration; ITL, intention to leave current position.

The ability of various EE cutoff values to identify concerning levels on the SHS, CES-D10, WLI, and ITL metrics is presented in Table 5. Results indicate that there is a large increase in CES-D10, WLI, and ITL scores when comparing those who endorse moderate vs. severe EE for both the EE9item and EE5item.^1^

**TABLE 5:** Proportion of respondents reporting concerning levels on the SHS, CES-D10, WLI, and ITL scales at various thresholds on the EE metrics.

	SHS score <5 (%)	CES-D10 score ≥10 (%)	WLI score >2 (%)	ITL score ≥50 (%)
**EE9item:**				
No EE	6.8	0.0	11.4	6.6
Mild EE	19.0	4.2	36.2	18.4
Moderate EE	48.7	28.2	58.4	55.0
Severe EE	68.6	70.3	84.1	82.1
**EE5item:**				
No EE	5.1	0.0	13.5	6.7
Mild EE	16.6	3.2	33.2	15.0
Moderate EE	42.9	17.4	52.4	47.0
Severe EE	60.0	52.7	78.6	70.6
**EE1item5pt:**				
No EE	9.5	1.9	24.0	12.3
Mild EE	23.8	4.5	38.0	14.4
Moderate EE	38.7	16.0	46.4	43.2
Severe EE	45.9	30.9	62.9	53.9
**EE1item7pt:**				
≥1 time/week	53.1	43.8	74.6	61.9

The cutoffs for concerning levels are SHS (<5) (Lyubomirsky and Lepper, 1999), CES-D10 (≥10) (Björgvinsson et al., 2013), WLI (>2) (Sexton et al., 2017; Schwartz et al., 2019), and ITL (≥50). For five-point agree-disagree metrics (EE9item, EE5item, EE1item5pt) scores of 0 = no EE, 0.01–49.9 = mild EE, 50–74.9 = moderate EE, and ≥75 = severe. For the EE1item7pt metric, a cutoff of “once a week or more” is often used to indicate a concerning level (West et al., 2009; Brady et al., 2020). EE9item, 9-item emotional exhaustion metric; EE5item, 5-item emotional exhaustion metric; EE1item5pt, single item emotional exhaustion metric using five-point agree-disagree scale; EE1item7pt, single item emotional exhaustion metric using 7-point frequency response scale; CES-D10, center for epidemiological studies depression scale 10-item; SHS, subjective happiness scale; WLI, work-life integration; ITL, intention to leave current position.

## Discussion

Concern about HCW emotional exhaustion due to the fast paced and high intensity nature of working in healthcare has been elevated by the COVID-19 pandemic (Sexton et al., 2022b; Shanafelt et al., 2022). Healthcare systems are increasingly motivated to assess and reduce the level of EE in the workforce. Having brief, no-cost, reliable and validated metrics of EE is essential to minimize respondent burden and facilitate inclusion in larger health system surveys evaluating a variety of safety culture, engagement, and wellbeing domains. This evaluation of the EE9item, EE5item, EE1item5pt, and EE1item7pt metrics in a large sample of HCW across the country revealed that the EE5item has strong psychometric reliability and construct validity. The EE1item5pt and EE1item7pt do not exhibit as strong convergent construct validity as the EE5item (i.e., they exhibited significantly weaker correlations to all other outcomes), however, in situations where a quick screening assessment is needed for a signal of EE, they can be used with relative confidence (with a slight advantage for EE1item5pt over EE1item7pt). A guide to using, scoring and interpreting the EE5item, including verbatim items, instructions and benchmarking results from a sample of over 30,000 healthcare workers can be found in the Supplementary material.

The EE5item is equivalent or superior to the EE9item in several ways. The internal reliability (alpha = 0.87) was only slightly lower than the EE9item (alpha = 0.91), despite the fact that higher numbers of items artificially inflate the alpha. The EE5item alpha was consistent with previous alphas reported (0.84 to 0.92) (Adair et al., 2018, 2020a,c; Sexton et al., 2018, 2022b; Schwartz et al., 2019; Sexton and Adair, 2019; Profit et al., 2021). These values rest solidly in the >0.80 range acceptable for implementing cutoff scores and border the >0.90 threshold for clinically important decisions (Nunnally, 1978; Nunnally et al., 1994). Cronbach’s alpha can be artificially inflated by either increasing the number of items so that the quantity overpowers the quality of items, or reducing the number of items in a scale to select only the most highly correlated items (Kopalle and Lehmann, 1997; Cho and Kim, 2015). Considering this, the CFA adds more granularity to how the two scales model the underlying construct of EE. The CFA fit indices for the EE5item indicated much better fit overall than for the EE9item, in which none of the indices indicated acceptable model fit (Browne and Cudeck, 1992; Hu and Bentler, 1999). The exploratory factor analysis (EFA) results demonstrate that EE emerges as a distinct construct from depression when using both the EE9item and EE5item (see Supplementary material). The EFA also demonstrated that the two items “working with people directly puts too much stress on me” and “working with people all day is really a strain for me,” loaded on their own factor. These items are part of the EE9item, but not included in the EE5item.

Previous EFAs and CFAs of the EE9item have shown the two “stress” and “strain” items to be particularly problematic, because they are very similar and locally dependent (Worley et al., 2008; Mukherjee et al., 2020). Both of these items also strongly load on the DP factor of burnout rather than EE (Poghosyan et al., 2009). Item response theory analyses also revealed that higher EE symptom severity is required to endorse the “stress” or “strain” items (a z-score > 1.57 standard deviations above the mean), or “I feel like I am at the end of my rope” (z-score > 1.0 standard deviations above the mean) (Brady et al., 2020). These “stress,” “strain,” and “rope” questions are not included in the EE5item. The high symptom severity required to endorse those EE9item questions also helps explain why the mean score is lower for the EE9item (31.6) than the EE5item (36.6). Analyses further demonstrated that the EE5item correlates as well as the EE9item with SHS, WLI, and CES-D10 and significantly stronger with ITL. It is overall more sensitive and less specific than the EE9item for detecting concerning levels on these other metrics, making it a useful screening tool.

The EE9item and EE5item predicted the 4 other wellbeing metrics better than the single item EE measures, and the EE5item was slightly better than the EE9item. Correlations among both single item measures and every other metric (SHS, CES-D10, WLI, and ITL) are significantly weaker than the EE9item and EE5item. This demonstrates the value of using an EE metric with multiple items to capture the full range of the underlying dimension of EE (Robinson, 2018). Although the 1-item questions can be administered to quickly gauge a signal of EE in a population, they do not detect these other important wellbeing outcomes as effectively.

The EE1item7pt had the lowest mean EE score (29.7), likely because most respondents (43.6%) endorsed feeling burned out “a few times a year or less,” the second lowest frequency, and those who selected lower frequencies had on average a higher agreement with EE on the EE5item and EE9item. The seven-point response scale inherently does not assess current burnout, but rather its frequency across a whole year. It is possible for a respondent to report burnout frequency as “a few times a year or less” yet be severely burned out when they complete the survey. This is an important consideration when selecting which metric to administer. The EE1item7pt also correlates weaker than the EE1item5pt to the EE5item (0.66 vs. 0.80, respectively) and the EE9item (0.67 vs. 0.81, respectively).

A variety of thresholds have been used for measurements of burnout and EE (Rotenstein et al., 2018), and they can be useful to identify a subset of respondents who may require additional support or intervention. Even without a consensus clinical definition for burnout syndrome, it is important to detect which HCWs may be dealing with more exhaustion and emotional burden at work, provide resources and track trends over time. Thresholds such as “% emotionally exhausted” are useful in debriefings and for summarizing results for those less accustomed to dealing with survey data. Individuals may find it easier to grasp that, for instance, 59% of their unit is experiencing emotional exhaustion, compared to hearing that the mean EE score for the group is 3.1. An alternative to thresholds is to use measures of central tendency, such as means, to track trends in data over time. This can also be valuable to assess how a population is changing, because respondents who score just two points away from each other might be identified as clinically different even though in reality their EE level is virtually the same. We recommend reporting the mean and the percent for clarity. According to the thresholds for EE used here, a *majority* of participants reporting severe EE on the EE9item *and* EE5item have concerning levels of depression, work-life imbalance, subjective happiness and ITL their current position.

Ultimately when deciding which metric for EE to administer, the EE5item has optimal psychometrics and convergent construct validity. Compared to the 9-item version, the EE5item is free to administer, less burdensome for respondents, has improved model fit for EE, and has either equivalent or superior correlation with other important metrics: SHS, CES-D10, WLI, and ITL. Particularly with a move among health systems to administer shorter surveys more frequently in “pulses” rather than longer surveys with more time between them, identifying valid and abbreviated EE metrics to gauge burnout is crucial. The questions retained in the EE5item (I feel burned out, fatigued, frustrated, working too hard) as well as the newly developed item (events at work) were chosen because they are more representative of the overall EE construct, as reflected in the improved CFA model fit compared to the EE9item. One thing to keep in mind is that respondents on average score slightly higher EE on the EE5item, likely because several items from the EE9item that require severe EE to endorse have been removed. For a screening tool, the results from the EE5item are the sought after criteria. The EE1item5pt and EE1item7pt do not have as strong convergent construct validity for other important metrics, but can be used to quickly assess or track trends in the EE level of a population.

This study is limited by the age of the dataset, which was collected in 2013. For this reason, the level of burnout in the sample is not timely (EE is worse now) (Sexton et al., 2022b). Nevertheless, the psychometric analyses and convergent validity correlations set forth as aims should not be affected by the year data were collected. Recent EE5item results from over 30,000 HCWs in 2021/2022 show the reliability of the scale was above 0.90 (Supplementary material). There is little reason to believe that relationships between items within scales or associations among the four EE metrics and other wellbeing outcomes would be significantly different based on the age of the data. Another limitation is that five-point response options were used for the EE9item, whereas the original 9-Item EE scale from the MBI uses seven-point response options. Nonetheless, all survey items were the same. The study’s respondents were from diverse healthcare settings across the United States, therefore the results may be fairly generalizable. However, given these participants were physicians, it is possible that the pattern of results could differ in other HCW roles, therefore the generalizability to other groups is not yet clear. The study is also limited by its use of self-report data which poses a risk of response, selection, and social desirability biases. The high response rate and use of psychometrically validated scales helped to minimize these biases. Finally, because this is a cross-sectional study, test–retest reliability could not be examined. Nevertheless, the responsiveness of the EE5item to interventions (Sexton and Adair, 2019; Adair et al., 2020a,c, 2022; Profit et al., 2021; Sexton et al., 2021a,2022a) and changing work stresses (Sexton et al., 2022b), strongly suggests that changes in scores very consistently move in the predicted direction and magnitude.

## Conclusion

There is considerable interest in assessing EE, a key component of HCW wellbeing, using brief and valid metrics. The current study evaluated the psychometric properties of four measures of EE (EE9item, EE5item, EE1item5pt, and EE1item7pt). The EE5item was equivalent or superior to the EE9item across analyses. EE5item has no cost, is easy to administer and interpret, and is a reliable metric for assessing EE. The decreased length reduces respondent burden and is of great value when assessing healthcare workers to make clinical and operational decisions. The lack of cost associated with this metric opens researchers to include a brief, reliable scale to assess emotional exhaustion during a time that wellbeing research is increasingly important. EE1item5pt and EE1item7pt did not correlate as well with happiness, depression, WLI, and ITL, however, they can serve as a proxy for the longer scales to detecting a signal of EE, when administering validated scales is not feasible.

## Data availability statement

The raw data supporting the conclusions of this article will be made available by the authors, without undue reservation.

## Ethics statement

The studies involving humans were approved by the Duke University Health System Institutional Review Board. The studies were conducted in accordance with the local legislation and institutional requirements. The Ethics Committee/Institutional Review Board waived the requirement of written informed consent for participation from the participants or the participants’ legal guardians/next of kin because of the low-risk level of the data and its deidentification.

## Author contributions

CP: Formal analysis, Investigation, Methodology, Writing – original draft, Writing – review and editing. KA: Formal analysis, Investigation, Methodology, Project administration, Supervision, Writing – review and editing. AF: Conceptualization, Data curation, Writing – review and editing. ML: Conceptualization, Writing – review and editing. JP: Data curation, Writing – review and editing. PM: Resources, Supervision, Writing – review and editing. JS: Conceptualization, Data curation, Investigation, Methodology, Supervision, Writing – review and editing.

## Footnotes

1 Divergent validity was assessed using Exploratory Factor Analyses (EFAs). Since depression is a similar but distinct construct from EE, we conducted two EFAs to test whether the depression items (CES-D10 items) loaded onto different factors than the items from the EE5item and EE9item. Indeed, both EFAs revealed that none of the depression items loaded on the same factor as any of the EE items, indicating discriminant validity. These results are described in detail in the Supplementary file (section VI).
