# Supplementary material for: A new look at an old well-being construct: evaluating the psychometric properties of 9, 5, and 1-item versions of emotional exhaustion metrics
Source: Front Psychol. 2023 Nov 23;14:1267660. doi: 10.3389/fpsyg.2023.1267660 (PMC10702494; doi:10.3389/fpsyg.2023.1267660)
Supplement: Supplementary file 1 [file Data_Sheet_1.docx]

Supplemental for

**A New Look at an Old Well-being Construct: Evaluating the Psychometric Properties of 9, 5, and 1-item Versions of Emotional Exhaustion Metrics.**

Table of Contents

1. Scoring for the 5-item Emotional Exhaustion Scale
2. Benchmarking for the 5-item Emotional Exhaustion Scale
3. Cronbach’s alpha and CFA for the benchmarking dataset for the 5-item Emotional Exhaustion Scale
4. Scoring for the 9-item Emotional Exhaustion Scale
5. Scoring for the two 1-item Emotional Exhaustion Measures
6. Table comparing all 4 metrics on structure, mean scores, alphas and psychometrics
7. Exploratory Factor Analyses for Discriminant Validity
8. Scoring for the 5-item Emotional Exhaustion Scale

**Emotional Exhaustion 5-item (EE5item)**

**Choose your responses using the scale below:**

| **A** | **B** | **C** | **D** | **E** | | **X** | | | | |
| --- | --- | --- | --- | --- | --- | --- | --- | --- | --- | --- |
| **Disagree Strongly** | **Disagree Slightly** | **Neutral** | **Agree Slightly** | **Agree Strongly** | | **Not Applicable** | | | | |
| 1. Events in this work setting affect my life in an emotionally unhealthy way. | | | | | A | B | C | D | E | X |
| 1. I feel burned out from my work. | | | | | A | B | C | D | E | X |
| 1. I feel fatigued when I get up in the morning and have to face another day on the job. | | | | | A | B | C | D | E | X |
| 1. I feel frustrated by my job. | | | | | A | B | C | D | E | X |
| 1. I feel I am working too hard on my job. | | | | | A | B | C | D | E | X |

**Suggested variable labels and SPSS code for scoring:**

| Variable name |  |
| --- | --- |
| EEevents | Events in this work setting affect my life in an emotionally unhealthy way. |
| EEburn | I feel burned out from my work. |
| EEexhausted | I feel fatigued when I get up in the morning and have to face another day on the job. |
| EEfrust | I feel frustrated by my job. |
| EEwork2hd | I feel I am working too hard on my job. |
|  | **SPSS code to compute total score (avg across items, - 1, x 25):**  **COMPUTE SCOREE= ((MEAN(EEevents, EEburn, EEexhausted, EEfrust, EEwork2hd))-1)*25.**  **VARIABLE LABELS SCOREE '5 Item Emotional Exhaustion Scale Score'.** |

This 5-item Emotional Exhaustion derivative scale has been shown to have excellent psychometric properties,^1–5^ external validity,^4,6,7^ and is responsive to interventions.^2,3,8,9^ Participants respond using a 5-point Likert scale (1 = disagree strongly; 5 = agree strongly). Each participant’s mean of the 5 items is converted to a 0-100 point scale, with higher scores representative of more severe burnout. Consistent with prior research, for 5-point agree-disagree scales a score of 0-49.9 indicates no EE (disagreeing to all items), 50-74.9 indicates mild EE (neutral or agreeing slightly, on average), 75 – 94.9 indicates moderate EE (agreeing slightly or strongly, on average), and ≥ 95 indicates severe EE (agreeing strongly to all items). A threshold of 50 or above for mild burnout was used to demonstrate prevalence and was used for grouping participants as “percent concerning”.^1,7,9,10^ The cutoff scores are not diagnostic, but rather is used to gauge the severity of EE present. The cutoffs provide an anchor for interpretation and a tool for communicating trends in the data.

1. Benchmarking for the 5-item Emotional Exhaustion Scale

As a current benchmark, we used a large-scale dataset containing the 5-item EE scale collected in September 2019, September 2020 and September 2021/January 2022 from US Healthcare Workers. A comparison of rates of EE in Healthcare workers before, and two times during the COVID-19 pandemic was published using these results in JAMA Network Open.^11^

The most recently collected wave (September 2021/January 2022 (N = 31,475)) offers an excellent benchmarking opportunity for researchers or patient safety professionals to compare any 5-item EE results. Below we outline the methods used for these datasets, as well as the benchmarking figures for use by others. Online PowerPoint distributions of these figures are available online with this supplemental so that you may insert where your group falls in the benchmarking.

Methods

Emotional exhaustion was assessed using routine electronic (via email and/or access to a generic link) administrations of the SCORE^1^ survey in two USA health systems across 76 widely geographically dispersed hospitals before the pandemic (September 2019), after the start of the pandemic (September 2020), and after vaccines/vaccine mandates/Delta variant (September 2021 in the first system and December 2021/January 2022 in the second system (N for this 3^rd^ wave = 31,475)). SCORE assesses safety culture, workforce well-being and engagement, including an emotional exhaustion (EE) scale because HCW well-being was increasingly recognized as common,^12,13^ expensive,^14^ and treatable.^15,16^ EE assesses the extent to which one feels drained, overwhelmed and unable to meet demands. Example items include “I feel frustrated by my job,” and “Events in this work setting affect my life in an emotionally unhealthy way.” The response scale for EE ranges from 1 (strongly disagree) to 5 (strongly agree), such that higher scores reflect higher levels of exhaustion.

These results were aggregated at the work-setting level to create the benchmarking distribution provided here. Specifically, we calculated the percent positive score, or the percent of those scoring at or above the threshold of 50 points on the 1-100 scale within each work setting. Work settings with fewer than 5 respondents were not included (n = 7) for reliability purposes.

*Supplemental Figure 1. Percent of Respondents Reporting Emotional Exhaustion (EE5item) across a Random Sample of 400 Work Settings*

1^st^

Quartile

2^nd^ Quartile

3^rd^ Quartile

4^th^ Quartile

Note: Each bar = 1 Work setting. Only work settings with 5 or more HCWs were included.

**% respondents reporting EE**

Each bar = 1 Work setting

1. Cronbach’s alpha and CFA for the benchmarking dataset for the 5-item Emotional Exhaustion Scale

We calculated the Cronbach’s alpha and examined the 1-factor EE Confirmatory Factor Analysis model with our most recent wave, i.e., the 2021/2022 dataset outlined above.

The Cronbach’s alpha for the 5-item EE scale in the 2021/2022 wave was .937.

For the CFA, we used the same fit indices criteria outlined in the main paper: Root mean square error approximation (RMSEA) with adequate fit < .08, Tucker-Lewis fit index (TLI) with adequate fit > .95, confirmatory fit index (CFI) with adequate fit > .95, and standardized root mean square residual (SRMR) with < .08 considered adequate fit.^17,18^

The more current dataset included de-identified work setting ID numbers for each respondent, allowing us to account for clustering at the work setting level. 1511 work settings were accounted for and 31,010 individuals were included in the analysis.

The CFA model revealed good fit for the EE5item scale for the 2021/22 data: RMSEA = .062 (90% CI .057 - .066), CFI = .997, TLI = .994, and SRMR = .011.

*EE5item CFA model with the 2021/22 data, with Standardized Factor Loadings*
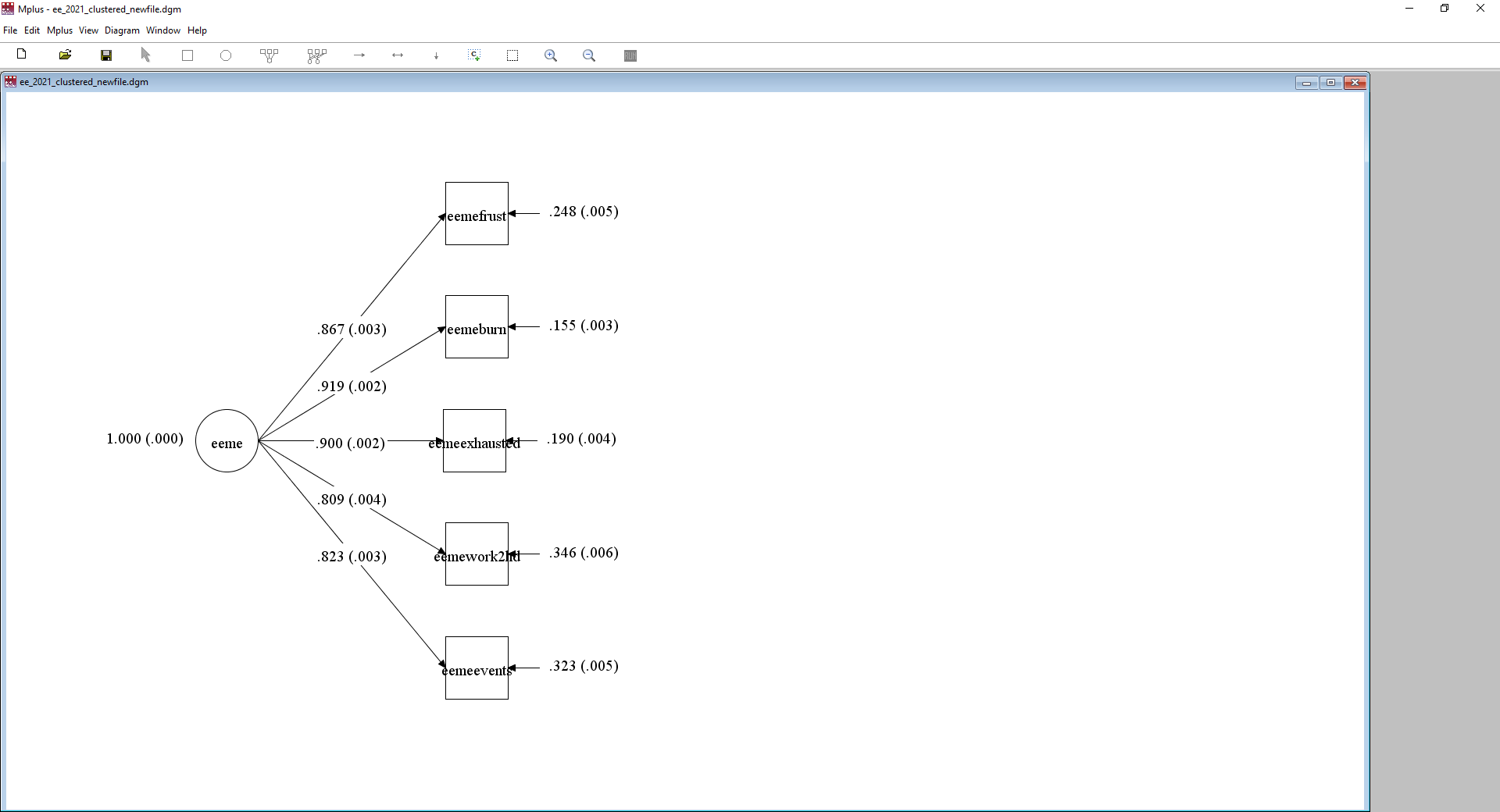


1. Scoring for the 9-item Emotional Exhaustion Scale

**Emotional Exhaustion 9-item (EE9item)**

**Choose your responses using the scale below:**

| **A** | **B** | **C** | **D** | **E** | | **X** | | | | |
| --- | --- | --- | --- | --- | --- | --- | --- | --- | --- | --- |
| **Disagree Strongly** | **Disagree Slightly** | **Neutral** | **Agree Slightly** | **Agree Strongly** | | **Not Applicable** | | | | |
| 1. I feel burned out from my work. | | | | | A | B | C | D | E | X |
| 1. I feel fatigued when I get up in the morning and have to face another day on the job. | | | | | A | B | C | D | E | X |
| 1. I feel frustrated by my job. | | | | | A | B | C | D | E | X |
| 1. I feel I am working too hard on my job. | | | | | A | B | C | D | E | X |
| 1. I feel emotionally drained from my work. | | | | | A | B | C | D | E | X |
| 1. I feel used up at the end of the workday. | | | | | A | B | C | D | E | X |
| 1. I feel like I am at the end of my rope. | | | | | A | B | C | D | E | X |
| 1. Working with people directly puts too much stress on me. | | | | | A | B | C | D | E | X |
| 1. Working with people all day is really a strain for me. | | | | | A | B | C | D | E | X |

**Suggested variable labels and SPSS code for scoring:**

| Variable name |  |
| --- | --- |
| EEMEburn | I feel burned out from my work. |
| EEMEexhausted | I feel fatigued when I get up in the morning and have to face another day on the job. |
| EEMEfrust | I feel frustrated by my job. |
| EEMEwork2hd | I feel I am working too hard on my job. |
| EEMEdrained | I feel emotionally drained from my work. |
| EEMEusedup | I feel used up at the end of the workday. |
| EEMErope | I feel like I am at the end of my rope. |
| EEMEstress | Working with people directly puts too much stress on me. |
| EEMEstrain | Working with people all day is really a strain for me. |
|  | **SPSS code to compute total score (avg across items, - 1, x 25):**  **COMPUTE SCOREEME= ((MEAN(EEMEevents, EEMEburn, EEMEexhausted, EEMEfrust, EEMEwork2hd, EEMEdrained, EEMEusedup, EEMErope, EEMEstress, EEMEstrain))-1)*25.**  **VARIABLE LABELS SCOREEME '9 Item Emotional Exhaustion Scale Score'.** |

The 9-item scale includes all questions from the Maslach Burnout Inventory (MBI) emotional exhaustion subscale.^19^ Compared to the other two subscales in the MBI, the EE subscale has been shown to produce the largest and most consistent internal reliability estimates,^20–22^ the highest test-retest reliability,^19^ and has been demonstrated to be the only subscale adequately precise for individual-level measurement.^23^ Instead of using the 7-point frequency scale used by the original MBI, we used a 5-point agree-disagree scale. Each participant’s mean of the 9 items was converted to a 0-100 point scale, with higher scores representative of more severe burnout. Consistent with prior research, for 5-point agree-disagree scales a score of 0-49.9 indicates no EE (disagreeing to all items), 50-74.9 indicates mild EE (neutral or agreeing slightly, on average), 75 – 94.9 indicates moderate EE (agreeing slightly or strongly, on average), and ≥ 95 indicates severe EE (agreeing strongly to all items). A threshold of 50 or above for mild burnout was used to demonstrate prevalence and was used for grouping participants as “percent concerning”.^1,7,9,10^

1. Scoring for the two 1-item Emotional Exhaustion Measures

**Emotional Exhaustion 1-item using a 5-point Scale (EE1item5pt)**

**Choose your responses using the scale below:**

| **A** | **B** | **C** | **D** | **E** | | **X** | | | | |
| --- | --- | --- | --- | --- | --- | --- | --- | --- | --- | --- |
| **Disagree Strongly** | **Disagree Slightly** | **Neutral** | **Agree Slightly** | **Agree Strongly** | | **Not Applicable** | | | | |
| 1. I feel burned out from my work. | | | | | A | B | C | D | E | X |

**Suggested variable labels and SPSS code for scoring:**

| Variable name |  |
| --- | --- |
| EEMEburn | I feel burned out from my work. |
|  | **SPSS code to compute total score (single item score, - 1, x 25):**  **COMPUTE EE1itemBurn= ((EE1itemBurn))-1)*25.**  **VARIABLE LABELS EE1itemBurn'Single Item Burned Out from Work'.** |

Each participant’s score for the single question was converted to a 0-100 point scale, with higher scores representative of more severe burnout. Consistent with prior research, for 5-point agree-disagree items a score of 0-25 indicates no EE (disagreeing), 50 indicates mild EE (neutral), 75 indicates moderate EE (agreeing slightly), and 100 indicates severe EE (agreeing strongly). A threshold of 50 or above for mild burnout was used to demonstrate prevalence and was used for grouping participants as “percent concerning”.^1,7,9,10^

**Emotional Exhaustion 1-item using a 7-point Scale (EE1item7pt)**

**Choose your responses using the scale below:**

| **A** | **B** | **C** | **D** | **E** | **F** | **G** | | | | **X** | | | | | |
| --- | --- | --- | --- | --- | --- | --- | --- | --- | --- | --- | --- | --- | --- | --- | --- |
| **Never** | **A few times a year or less** | **Once a month or less** | **A few times a month** | **Once a week** | **A few times a week** | **Every day** | | | | **Not Applicable** | | | | | |
| 1. How often I feel burned out from my work. | | | | | | | A | B | C | | D | E | F | G | X |

Responses to this item were averaged and converted to a 0-100 scale to allow comparison to the 5-point scales. For the EE1item7pt metric, a cutoff of “once a week” or more is often used to indicate concerning levels.^23–25^ A score of 66.67 out of 100 indicates “once a week.” Please see West et al. for scoring details.^24^

VI. Supplemental Table 1. EE9item, EE5item, EE1item5pt, and EE1item7pt items, mean scores, Cronbach alpha, and CFA fit indices

| **Abbreviated Name** | **Items Included** | **Response Scale^a^** | **Mean score (95% CI)** | **Cronbach Alpha^b^** | **CFA fit indices^c^** |
| --- | --- | --- | --- | --- | --- |
| **EE9item** | 1. fatigued 2. burned out 3. frustrated 4. working too hard 5. emotionally drained 6. used up 7. at the end of my rope 8. too much stress 9. a strain for me | 5-point (Disagree Strongly to Agree Strongly) | 31.6 (30.3 - 32.8) | 0.91 | RMSEA = .219 (90% CI .211 - .228)  CFI = .790  TLI = .720  SRMR = .088 |
| **EE5item Current Issue** | 1. fatigued 2. burned out 3. frustrated 4. working too hard 5. events at work | 5-point  (Disagree Strongly to Agree Strongly) | 36.6 (35.2 – 38.0) | 0.87 | RMSEA = .082 (90% CI .063 - .103)  CFI = .986  TLI = .972  SRMR = .018 |
| **EE5item**  **Jama Network Open 2022 (data from 2021)** | 1. fatigued 2. burned out 3. frustrated 4. working too hard 5. events at work | 5-point  (Disagree Strongly to Agree Strongly) | 43.2 (42.9 – 43.6) | 0.93 |  |
| **EE1item5pt** | 1. burned out | 5-point  (Disagree Strongly to Agree Strongly) | 34.3 (32.6 – 36.0) |  |  |
| **EE1item7pt** | 1. burned out | 7-point (Never to Every Day) | 29.7 (28.4 – 31.0). |  |  |

Note: **^a^**5-point scales options include: 1) disagree strongly, 2) disagree slightly, 3) neutral, 4) agree slightly, and 5) agree strongly, while 7-point scale options include: 1) never, 2) a few times a year or less, 3) a few times a month, 4) once a week, 5) a few times a week, 6) every day. ^b^ Cronbach’s alpha of at least .70 is acceptable for early-stage research, .80 for implementing cutoff scores, and .90 if clinically important decisions are being made.^26,27^ ^c^ CFA indices considered adequate fit are RMSEA < .08, TLI > .95, CFI > .95, and SRMR < .08. ^17,18^

1. Exploratory Factor Analyses for Discriminant Validity

Two Exploratory Factor Analyses (EFAs) were conducted to test for discriminant validity for both the 5- and 9-item EE scales. The Center for Epidemiological Studies Depression Scale 10-item (CES-D10) was selected to serve as a similar, but different construct to test against for discriminant validity. Both EFAs used Maximum Likelihood for the extraction method and Direct Oblimin for the rotation. Factor selection was made based on the Scree Plot and the number of Eiganvalues > 1.

The EFA of the 5-item EE scale items and the CES-D10 items revealed 3 factors. Analysis of the Pattern Matrix (see below) revealed that all but two of the CES-D10 items loaded on to the first factor. These two items loaded on to their own factor and they are only items that are reverse scored. Reverse scored items are often included in scales to help participants pay attention, however they can result in patterns of responses unlike the rest of the scale. Indeed, prior research has shown that reverse scored items often behave differently due to method effects and result in multidimensionality.^28, 29^ All of the 5-item EE items loaded cleanly on to their own factor.

The EFA of the 9-item EE scale and the CES-D10 revealed 4 factors. Analysis of the Pattern Matrix (see below) revealed that most but not all of the EE items loaded onto the first factor. The item “I’m at the end of my rope” did not load strongly onto any factor, but its .337 loading was included in the first factor. The last two EE items (‘people stress’ and ‘people strain’) loaded on to their own factor. Otherwise, the CES-D10 items behaved similarly as they did in the prior EFA; all but the reverse scored items loaded on to their own factor and the reverse scored items loaded on to their own factor.

Taken together, these results indicate discriminant validity for the EE scales. None of the CES-D10 items loaded on to the same factor as the EE scale. This was the case for both the 5 and 9-item EE scale.

For the 5-item EE EFA, all 5 of the EE items loaded on the same factor. However, not all 9 EE items loaded on to the same factor. The “rope” item did not load strongly onto any factor, and the “people stress” and “people strain” items loaded onto their own factor. This appears in line with the poorer fit indices obtained in the CFA for the 9-item EE instrument.

| **Pattern Matrix for 5-item EE scale and CES-D Scale^a^** | | | |  |
| --- | --- | --- | --- | --- |
|  | Factor | | |  |
|  | 1 | 2 | 3 |  |
| EEfatigue | 0.175 | -0.607 | -0.002 |  |
| EEburnout | 0.005 | -0.874 | -0.057 |  |
| EEfrustrated | -0.032 | -0.821 | -0.066 |  |
| EEwork2hard | -0.053 | -0.752 | 0.051 |  |
| EEevents | 0.207 | -0.553 | -0.125 |  |
| CESD1repre | 0.572 | -0.178 | -0.039 |  |
| CESD2repre | 0.575 | -0.113 | 0.081 |  |
| CESD3repre | 0.716 | 0.017 | -0.146 |  |
| CESD4repre | 0.625 | -0.089 | 0.002 |  |
| CESD5repre* | 0.060 | 0.003 | 0.675 |  |
| CESD6repre | 0.663 | 0.069 | -0.064 |  |
| CESD7repre | 0.564 | -0.073 | 0.106 |  |
| CESD8repre* | -0.121 | 0.111 | 0.651 |  |
| CESD9repre | 0.663 | 0.134 | -0.074 |  |
| CESD10repre | 0.626 | -0.067 | 0.081 |  |
| Extraction Method: Maximum Likelihood.   Rotation Method: Oblimin with Kaiser Normalization. | | | |  |
| 1. Rotation converged in 7 iterations. 2. *reverse scored item | | | |  |

**Scree Plot for Pattern Matrix for 5-item EE scale and CES-D Scale**


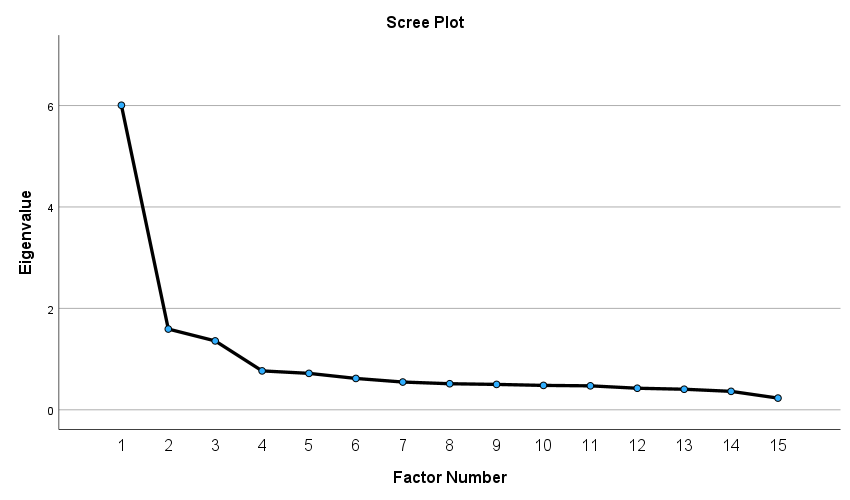


| **Pattern Matrix for 9-item EE scale and CES-D Scale^a^** | | | | |
| --- | --- | --- | --- | --- |
|  | Factor | | | |
|  | 1 | 2 | 3 | 4 |
| EEfatigue | 0.533 | -0.059 | 0.201 | -0.016 |
| EEburnout | 0.775 | -0.037 | 0.062 | -0.095 |
| EEfrustrated | 0.757 | 0.008 | 0.023 | -0.104 |
| EEwork2hard | 0.809 | 0.034 | -0.053 | 0.037 |
| EEdrained | 0.878 | -0.034 | -0.019 | -0.014 |
| EEusedup | 0.816 | -0.059 | -0.009 | 0.063 |
| EErope | 0.337 | -0.220 | 0.234 | -0.220 |
| EEpeoplestress | -0.053 | -1.001 | -0.039 | -0.002 |
| EEpeoplestrain | 0.058 | -0.845 | 0.022 | 0.023 |
| CESD1repre | 0.146 | -0.026 | 0.587 | -0.051 |
| CESD2repre | 0.063 | -0.057 | 0.584 | 0.090 |
| CESD3repre | -0.035 | 0.005 | 0.734 | -0.163 |
| CESD4repre | 0.073 | -0.047 | 0.616 | -0.001 |
| CESD5repre* | -0.005 | -0.005 | 0.060 | 0.656 |
| CESD6repre | -0.076 | -0.010 | 0.665 | -0.062 |
| CESD7repre | 0.115 | 0.035 | 0.562 | 0.115 |
| CESD8repre* | -0.091 | 0.035 | -0.123 | 0.634 |
| CESD9repre | -0.115 | -0.024 | 0.637 | -0.067 |
| CESD10repre | 0.061 | 0.002 | 0.636 | 0.083 |
| Extraction Method: Maximum Likelihood.   Rotation Method: Oblimin with Kaiser Normalization. | | | | |
| 1. Rotation converged in 5 iterations. 2. *reverse scored item | | | | |

**Scree Plot for Pattern Matrix for 9-item EE scale and CES-D Scale**


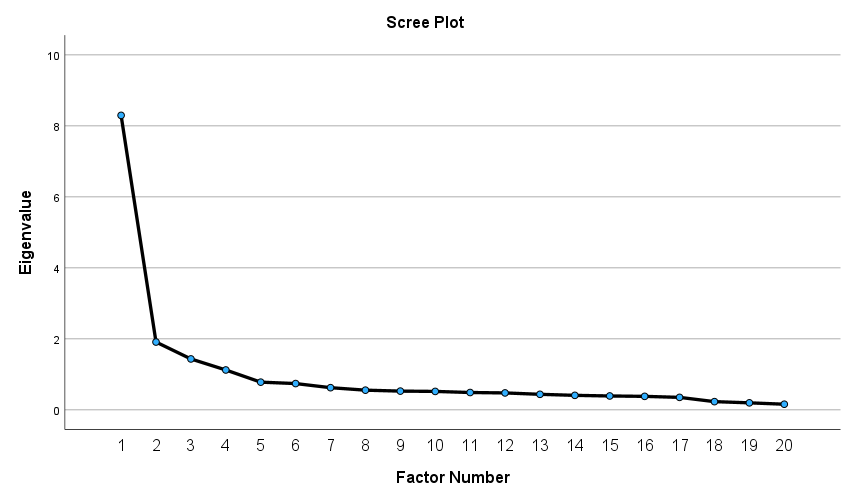


References

1. Sexton JB, Adair KC, Leonard MW, et al. Providing feedback following Leadership WalkRounds is associated with better patient safety culture, higher employee engagement and lower burnout. *BMJ Qual Saf*. 2018;27:261-270. doi:10.1136/bmjqs-2016-006399

2. Adair KC, Kennedy LA, Sexton JB. Three Good Tools: Positively reflecting backwards and forwards is associated with robust improvements in well-being across three distinct interventions. *J Posit Psychol*. Published online July 9, 2020:1-10. doi:10.1080/17439760.2020.1789707

3. Adair KC, Rodriguez-Homs LG, Masoud S, Mosca PJ, Sexton JB. Gratitude at Work: Prospective Cohort Study of a Web-Based, Single-Exposure Well-Being Intervention for Health Care Workers. *J Med Internet Res*. 2020;22(5):e15562. doi:10.2196/15562

4. Sexton JB, Schwartz SP, Chadwick WA, et al. The associations between work–life balance behaviours, teamwork climate and safety climate: cross-sectional survey introducing the work–life climate scale, psychometric properties, benchmarking data and future directions. *BMJ Qual Saf*. 2017;26(8):632-640. doi:10.1136/bmjqs-2016-006032

5. Sexton JB, Adair KC, Profit J, et al. Perceptions of Institutional Support for “Second Victims” Are Associated with Safety Culture and Workforce Well-Being. *Jt Comm J Qual Patient Saf*. 2021;47(5):306-312. doi:10.1016/j.jcjq.2020.12.001

6. Adair KC, Quow K, Frankel A, et al. The Improvement Readiness scale of the SCORE survey: a metric to assess capacity for quality improvement in healthcare. *BMC Health Serv Res*. 2018;18(1). doi:10.1186/s12913-018-3743-0

7. Schwartz SP, Adair KC, Bae J, et al. Work-life balance behaviours cluster in work settings and relate to burnout and safety culture: a cross-sectional survey analysis. *BMJ Qual Saf*. 2019;28(2):142-150. doi:10.1136/bmjqs-2018-007933

8. Sexton JB, Adair KC. Forty-five good things: a prospective pilot study of the Three Good Things well-being intervention in the USA for healthcare worker emotional exhaustion, depression, work-life balance and happiness. *BMJ Open*. 2019;9(3):e022695. doi:10.1136/bmjopen-2018-022695

9. Sexton JB, Adair KC, Profit J, et al. Safety Culture and Workforce Well-Being Associations with Positive Leadership WalkRounds. *Jt Comm J Qual Patient Saf*. 2021;47(7):403-411. doi:10.1016/j.jcjq.2021.04.001

10. Sexton JB, Adair KC, Proulx J, Profit J, Cui X, Bae J. Emotional Exhaustion Among U.S. Healthcare Workers Before and During the Covid-19 Pandemic, 2019-2021. *JAMA Netw Open*. In press.

11. Sexton JB, Adair KC, Proulx J, et al. Emotional Exhaustion Among US Health Care Workers Before and During the COVID-19 Pandemic, 2019-2021. *JAMA Netw Open*. 2022;5(9):e2232748. doi:10.1001/jamanetworkopen.2022.32748

12. Poghosyan L, Clarke SP, Finlayson M, Aiken LH. Nurse Burnout and Quality of Care: Cross-National Investigation in Six Countries. *Res Nurs Health*. 2010;33(4):288-298. doi:10.1002/nur.20383

13. Shanafelt TD, West CP, Sinsky C, et al. Changes in Burnout and Satisfaction With Work-Life Integration in Physicians and the General US Working Population Between 2011 and 2017. *Mayo Clin Proc*. 2019;94(9):1681-1694. doi:10.1016/j.mayocp.2018.10.023

14. Han S, Shanafelt TD, Sinsky CA, et al. Estimating the Attributable Cost of Physician Burnout in the United States. *Ann Intern Med*. 2019;170(11):784-790. doi:10.7326/M18-1422

15. Sexton JB, Adair KC. Forty-five good things: a prospective pilot study of the Three Good Things well-being intervention in the USA for healthcare worker emotional exhaustion, depression, work-life balance and happiness. *BMJ Open*. 2019;9(3):e022695. doi:10.1136/bmjopen-2018-022695

16. Adair KC, Kennedy LA, Sexton JB. Three Good Tools: Positively reflecting backwards and forwards is associated with robust improvements in well-being across three distinct interventions. *J Posit Psychol*. Published online July 9, 2020:1-10. doi:10.1080/17439760.2020.1789707

17. Hu L tze, Bentler PM. Cutoff criteria for fit indexes in covariance structure analysis: Conventional criteria versus new alternatives. *Struct Equ Model Multidiscip J*. 1999;6(1):1-55. doi:10.1080/10705519909540118

18. Browne MW, Cudeck R. Alternative Ways of Assessing Model Fit. *Sociol Methods Res*. 1992;21(2):230-258. doi:10.1177/0049124192021002005

19. Maslach C, Jackson S, Leiter M. The Maslach Burnout Inventory: Third Edition. In: *Evaluating Stress: A Book of Resources*. ; 1997:191-218.

20. Wheeler DL, Vassar M, Worley JA, Barnes LLB. A Reliability Generalization Meta-Analysis of Coefficient Alpha for the Maslach Burnout Inventory. *Educ Psychol Meas*. 2011;71(1):231-244. doi:10.1177/0013164410391579

21. Loera B, Converso D, Viotti S. Evaluating the psychometric properties of the Maslach Burnout Inventory-Human Services Survey (MBI-HSS) among Italian nurses: how many factors must a researcher consider? *PloS One*. 2014;9(12):e114987. doi:10.1371/journal.pone.0114987

22. Kleijweg JHM, Verbraak MJPM, Van Dijk MK. The clinical utility of the Maslach Burnout Inventory in a clinical population. *Psychol Assess*. 2013;25(2):435-441. doi:10.1037/a0031334

23. Brady KJS, Ni P, Sheldrick RC, et al. Describing the emotional exhaustion, depersonalization, and low personal accomplishment symptoms associated with Maslach Burnout Inventory subscale scores in US physicians: an item response theory analysis. *J Patient-Rep Outcomes*. 2020;4(1):42. doi:10.1186/s41687-020-00204-x

24. West CP, Dyrbye LN, Sloan JA, Shanafelt TD. Single item measures of emotional exhaustion and depersonalization are useful for assessing burnout in medical professionals. *J Gen Intern Med*. 2009;24(12):1318-1321. doi:10.1007/s11606-009-1129-z

25. Li-Sauerwine S, Rebillot K, Melamed M, Addo N, Lin M. A 2-Question Summative Score Correlates with the Maslach Burnout Inventory. *West J Emerg Med*. 2020;21(3):610-617. doi:10.5811/westjem.2020.2.45139

26. Nunnally JC. *Psychometric Theory*. McGraw-Hill; 1978.

27. Nunnally J, Jum N, Bernstein IH, Bernstein I. *Psychometric Theory*. McGraw-Hill Companies,Incorporated; 1994.

28. Zhang, X., Noor, R., & Savalei, V. (2016). Examining the Effect of Reverse Worded Items on the Factor Structure of the Need for Cognition Scale. *PloS one*, *11*(6), e0157795. https://doi.org/10.1371/journal.pone.0157795

29. Bores, D. A., Vigneau, F., Lalande, F. (2006). Measuring the need for cognition: item polarity, dimensionality, and the relation with ability. Personality and Individual Differences, 40(4), 810-828. https://doi.org/10.1016/j.paid.2005.09.007.
